# Implementation and evaluation of an interprofessional simulation-based education program for undergraduate nursing students in operating room nursing education: a randomized controlled trial

**DOI:** 10.1186/s12909-015-0400-8

**Published:** 2015-07-09

**Authors:** Rongmei Wang, Nianke Shi, Jinbing Bai, Yaguang Zheng, Yue Zhao

**Affiliations:** 1 School of Nursing, Tianjin Medical University, Tianjin, China; 2 School of Basic Medicine, Tianjin Medical University, Tianjin, China; 3 School of Nursing, University of North Carolina, Chapel Hill, NC USA; 4 School of Nursing, University of Pittsburgh, Pittsburgh, PA USA

**Keywords:** Operating room nursing, Interprofessional, Simulation, Cooperative behavior

## Abstract

**Background:**

The present study was designed to implement an interprofessional simulation-based education program for nursing students and evaluate the influence of this program on nursing students’ attitudes toward interprofessional education and knowledge about operating room nursing.

**Methods:**

Nursing students were randomly assigned to either the interprofessional simulation-based education or traditional course group. A before-and-after study of nursing students’ attitudes toward the program was conducted using the Readiness for Interprofessional Learning Scale. Responses to an open-ended question were categorized using thematic content analysis. Nursing students’ knowledge about operating room nursing was measured.

**Results:**

Nursing students from the interprofessional simulation-based education group showed statistically different responses to four of the nineteen questions in the Readiness for Interprofessional Learning Scale, reflecting a more positive attitude toward interprofessional learning. This was also supported by thematic content analysis of the open-ended responses. Furthermore, nursing students in the simulation-based education group had a significant improvement in knowledge about operating room nursing.

**Conclusions:**

The integrated course with interprofessional education and simulation provided a positive impact on undergraduate nursing students’ perceptions toward interprofessional learning and knowledge about operating room nursing. Our study demonstrated that this course may be a valuable elective option for undergraduate nursing students in operating room nursing education.

## Background

The operating room (OR) is a dynamic, high-risk setting requiring effective teamwork for the safe delivery of care. It brings together a diverse group of professionals who must work as a team to provide care to a surgical patient. Although effective operating room teamwork is a critical component for the safe delivery of care, its implementation in practice is far from ideal [1]. A major contributor to the inadequate teamwork of ORs is a culture characterized by interprofessional friction, a “silo mentality,” and “tribalism” among the professions [2]. One approach to counteracting negative cultural traits within the OR is to increase opportunities for positive practice and model effective team-based competencies by students in an interprofessional education (IPE) setting [3].

IPE, which is defined as “when two or more professions learn with, from and about each other to improve collaboration and the quality of care” [4], is a crucial pedagogical approach for preparing nursing and medical students to provide patient care in a collaborative team atmosphere. It has been demonstrated that IPE could be a central component in the provision of safe and quality patient care [5, 6]. Simulation based on real-world situations, encouraging critical thinking and decision making with team members from different disciplines, is one teaching strategy that supports interdisciplinary learning and is an attractive and feasible methodology for IPE [7]. Currently, simulation-based interprofessional activities are encountered in rapid response, trauma, and ICU training [8–10].

With the aim of extending the learning outcomes from the acquisition of essential clinical knowledge and skills to include communication and teamwork in the education of OR Nursing, an IPE program incorporating simulation was developed by our team. This interprofessional simulation-based education (IPSE) program brings together two recent trends in healthcare professional education: IPE and simulation. In this program, third-year nursing students and fourth-year medical students from our university were recruited and worked together for a joint learning experience involving a simulated OR in which the students functioned as a team to perform operations on experimental animals. The present study was designed to implement IPSE program and evaluate the influence of this program on nursing students’ attitudes toward IPE and knowledge of OR nursing.

## Methods

### Study design

A randomized controlled trial was conducted to evaluate the effects of a simulation-based interprofessional education program on nursing students’ communication, team-working, and clinical knowledge in operating room nursing.

### Samples and setting

A total of 55 third-year nursing students from Tianjin Medical University (Tianjin, China) were enrolled in this study. In order to prevent sampling bias, a random number table was used to assign the nursing students to the IPSE group (*n* = 28) or traditional course group (*n* = 27). Nursing students from both groups were female, aged 20–22 years (Mean age = 21), and without any previous experiences of a IPE or IPSE program. All nursing students who participated had just completed the Operating Room Nursing module. A total of 46 fourth-year medical students from the same university also participated in the study; they were in the Basic Surgical Techniques (BST) course, which was an established component of the medical curriculum. Arrangements were made so that the nursing students could join this activity.

In the IPSE group, 1–2 nursing students and 3–4 medical students were arranged into one group and were involved in the simulation scenario; they were assigned to perform surgical procedures as a team on anaesthetized animals. In the traditional course group, nursing students were instructed to practice operating room nursing skills under the supervision of an experienced instructor in the simulated operating room.

### Ethical considerations

Approval was obtained from the Research Ethics Committee of Tianjin Medical University. Participation in the evaluation was voluntary, consent forms were signed, and anonymity was guaranteed. Large animal models have been widely used in the training program based on surgical protocols [11], and animal manipulation was approved by animal committee of Tianjin Medical University from the perspective of the animal’s welfare.

### IPSE implementation

Three simulation scenarios including appendectomy, splenectomy, and small bowel resection and anastomosis were used in the IPSE group. Each simulation scenario went for three hours and occurred weekly over a two-week period in a simulated operating room. All students participated in two scenarios. During these scenarios, nursing students acted as scrub or circulating nurses, and medical students acted as surgeons. Prior to commencing the scenarios, a brief orientation was conducted that included an overview of the training goals and objectives, as well as a review of simulation ground rules and equipment.

During the operation, working as a scrub nurse, nursing students prepared the instruments, trolleys, and sterile supplies needed for the surgery, maintained a sterile environment, and provided skilled assistance to the surgeon during the operation. While working as a circulating nurse, nursing students were responsible for managing nursing care within the operating theatre and coordinating the needs of the surgical team with other care providers as needed for the completion of surgery. During the operations, students were entirely interactive with each other, exploring their capabilities, learning from each other, and helping each other when needed. The supervisors were there to aid students at a basic level and to facilitate advancement through successive stages of skills acquisition. These clinical-related scenarios were developed and reviewed by our IPSE team of Tianjin Medical University faculty members, which included representatives from nursing and medical schools. This faculty team ensured that the simulation scenarios incorporated aspects and problems important across the spectrum of professions.

### Measurements

The Readiness for Interprofessional Learning Scale (RIPLS) designed by Parsell and Bligh was used to measure the attitudes toward interprofessional teams and readiness for interprofessional education [12]. This questionnaire was completed before and after the course activity. It consists of 19 statements measuring the strengths of student’s beliefs concerning shared learning. The statements in the questionnaire are based on the desired or intended positive outcomes of successful shared learning. Each statement was scored on a 5-point Likert scale with anchors from 1 (*completely disagree*) to 5 (*completely agree*). Specifically, items 1–9 were labeled “teamwork and collaboration,” items 10–16 were labeled “professional identity,” and items 17–19 were labeled “roles and responsibilities.”

In the original English version, the RIPLS was found to be valid and reliable [12]. This scale has excellent reliability with a Cronbach’s alpha of 0.90 [12]. Subsequent studies using healthcare professions have also found the RIPLS to demonstrate acceptable levels of validity and reliability [13–15]. As no Chinese version was available, the English version of the RIPLS was translated into Chinese. Then, the Chinese version was back-translated into English by a Chinese-English speaking individual. Afterward, the two English translations were compared to distinguish the changes in the meanings. The study team and one specialist in psychometrics reviewed all the translation and cultural adaptation processes and corrected the changes. To ensure cultural equivalence, a panel of five experts confirmed the content validity of the Chinese version. The content validity index of RIPLS_Chinese_ was excellent at 0.91. Additionally, in our study, the Cronbach’s alpha coefficient was reported as follows: RIPLS (0.92), teamwork and collaboration (0.86), professional identity (0.80), and roles and responsibility (0.71).

As the study purpose was to explore and describe emergent themes rather than merely quantify response, it was considered appropriate to include additional open-ended questions that would provide greater depth of information and enable students to explain and qualify the choices they had made. The open-ended question asked students to provide their perceptions of the ISPE program workshops, including the acquisition of OR nursing knowledge and attitudes to shared learning. Stronach and Mclure [16] argued that the value of questionnaire data, and particularly small sample data, is improved by including such an opportunity.

Then, thematic content analysis, using word-processing tools and the principles of grounded theory, were used for the open-ended responses [17, 18]. Initial line-by-line analysis was followed by focused coding and recoding by constant comparison to produce themes and subthemes that emerged inductively from the data. The data were reviewed and coded independently by two authors to ensure reliability. Discrepancies were resolved by discussions to reach a consensus on different items.

The 20-item questionnaire was used to explore the nursing students’ knowledge about infection control, patient safety, quality assurance, and professional accountability in OR nursing. This was constructed based on the literature review and expert opinion by the research team. After the IPSE or control program, nursing students were asked to complete the questionnaire anonymously. The primary investigator (RW) of this study tested the linguistic clarity of the questions and the accuracy of alternative responses to this survey. Then, five experts confirmed that the content validity and content validity index of the questionnaire was 0.88. It had good reliability with a Cronbach’s alpha of 0.86. This test score ranges from 0 to 100. Higher scores indicated a higher-knowledge level in OR nursing.

The following are example questions from this knowledge questionnaire.

1. To prevent air contamination during a surgical procedure, the nurse can:
  A. Make sure everyone who enters the operating room is wearing a mask
  B. Disinfect the room following the surgical procedure
  C. Spray a disinfectant before and after surgery
  D. Ensure the doors to the operating suite are kept closed and avoid excessive traveling between rooms
2. With color change sterilization indicators, the white stripes on the tape change to _____ when the appropriate temperature has been met.
  A. Red
  B. Black
  C. Blue
  D. Green
3. Choose the best answer. A circulating nurse is removing used instruments from the OR and taking them to another room for decontamination. To maintain asepsis, she/he should:
  A. Cover the instruments prior to removing them
  B. Wear a mask when removing them
  C. Place them on a cart to avoid handling them
  D. Wear sterile gloves when removing them

### Data analysis

The Wilcoxon signed-rank test was used to analyze the differences in the individual question responses of nursing students’ attitudes toward IPE before and after the IPSE program. After the course, the differences in the nursing students’ knowledge about OR nursing between the IPSE and traditional course group were analyzed using independent samples *t*-tests. A *P* value less than 0.05 was considered statistically significant. Statistical analyses were performed using SPSS Statistics for Windows, Version 17.0 (SPSS, Chicago, IL, USA). Participant responses to the open-ended question were analyzed using qualitative methods.

## Results

### Readiness for interprofessional learning scale

A positive response to Questions 1–9 and 13–16 is associated with a positive attitude to IPE, and a negative response to Questions 10–12 and 17–19 is associated with a negative attitude to IPE. No significant difference in the responses to questions on the RIPLS was found between nursing students from the traditional course group and IPSE group before intervention. However, in nursing students from the IPSE group, there was a significant difference in the post-intervention questionnaire for Questions 3 (*p = 0*.046), 7 (*p =* 0.040), 13 (*p =* 0.023) and 14 (*p = 0*.013), which reflects more positive responses (Table 1). These results demonstrated the improved attitudes toward teamwork and collaboration, and professional identity after the IPSE course. In addition, although the difference between the pre- and post-questionnaire responses did not reach significance for the other questions, no question reflected a significantly more negative attitude to IPE in the post-intervention questionnaire.

**Table 1:** RIPLS questions with significant changes after student participation in the IPSE course

	Pre-intervention^a^	Post-intervention^a^	*P* value
*Teamwork and collaboration*			
Shared learning with other health care students will increase my ability to understand clinical problems.	4.35 ± 0.61	4.74 ± 0.48	0.046
For small-group learning to work, students need to trust and respect each other.	4.10 ± 0.69	4.42 ± 0.64	0.040
*Professional identity*			
Shared learning with other health care professionals will help me to communicate better with patients and other professionals.	4.22 ± 0.48	4.74 ± 0.45	0.023
I would welcome the opportunity to work on small group projects with other health care students.	4.16 ± 0.58	4.58 ± 0.51	0.013

^a^Data are given as Mean ± SD

### Responses to open-ended question

From the participants’ responses it is evident that they highly valued the IPSE experience. Qualitative analysis of the IPSE experience revealed four themes: communication with medical students, role awareness, a better way of learning, and future IPSE.

#### Communication with medical students

Nursing students emphasized the importance of communication between team members. One student stated, “*Some situations cannot flow smoothly when people do not communicate.*” Another commented, “*I now realize that nursing students and medical students have the same issues when communicating with each other.*”

#### Role awareness

Talking about role awareness, a student commented that the IPSE workshop “*enabled us to understand both our own role in relation to OR nursing care and also that of the other health care professionals*.” Another considered that it helped understand “*professional boundaries*.”

#### A better way of learning

Discussing the theme of a better way of learning, one student commented that learning in a simulated interprofessional setting was “*a better way of learning, because it provided a safe environment in which this learning could occur, and improved our essential practice-based skills*.” This was also revealed in student comments such as, “*Studying in this setting can transfer knowledge from scenarios to clinical situations, and facilitate working together for the greatest outcome for the patient.*”

#### Future IPSE

The theme of future IPSE was revealed in student comments such as, “*I felt I learned more from the collaboration with medical students. I thought I can do my best if I had more chance to practice my nursing skills like this*.”

### OR nursing knowledge questionnaire

On the total sum knowledge scores, nursing students in the IPSE group showed significantly higher scores (Mean [SD]: 83.50 [8.45]) compared to those in the traditional course group (Mean [SD]: 77.00 [7.33]; *p* < 0.05) (Table 2). The findings suggest that for these two groups of nursing students, there were differences in the level of knowledge of OR nursing after the IPSE or control program.

**Table 2:** Comparison of knowledge scores in nursing students from IPSE and traditional course groups

	Number	Mean ± SD	P value
IPSE group	28	83.50 ± 8.45	0.01
Traditional course group	27	77.00 ± 7.33	

## Discussion

Interprofessional communication is an important aspect of modern medical care. However, most current educational models focus on uniprofessional training, rather than truly interprofessional ones. The move from silo-based training models to true interprofessional models requires new attitudes and innovative tools [17, 19]. Both IPE and simulation are well-established tools in their own right for teaching and learning [7, 20]. However, to date, both have been predominantly used within postgraduate training programs [20]. The present study created a program that aimed to introduce the simulation-based IPE into pre-qualification education for health-care professional students. Our results suggest that the integration of the innovative stimulation-based experience with an IPE program not only improved nursing students’ attitude toward interprofessional working, but it also increased their OR nursing knowledge level.

Although effective operating room teamwork is essential for the safe delivery of care, its implementation in practice is far from ideal [1]. A contribution to counteracting negative cultural traits within the OR may be made by increasing opportunities for positive practice and modeling of effective team-based competencies by students in an IPE setting [21]. There is increased emphasis on the need to produce “safe” practitioners in increasing numbers while, at the same time, there are increasing restrictions on practicing on real patients [22]. Simulation-based learning can help to overcome this problem by providing students with an opportunity to apply learned concepts and skills in a realistic clinical setting [8, 23]. This provides a controlled educational environment that offers a safe place for experimentation and practice [8, 20, 24]. The innovation in the present study was designed to allow nursing and medical students to interact with each other at an early stage in their education through the joint simulation exercise. This allowed participants to focus on the specific skills sets related to communication and teamwork within a safe environment.

Creating an opportunity for these positive IPSE learning experiences is critical to change the destructive misperceptions harbored by each profession within the OR [25, 26]. As evidenced by our report, improved attitudes toward teamwork and collaboration, and professional identity were the pivotal products of this innovative undertaking, which imply a greater willingness to work together and may help reduce professional “uniqueness.” These results suggest an improvement in students’ attitudes toward interprofessional collaboration, which supports results seen in IPE training experiences in the literature [27, 28]. The data obtained in quantitative analysis were also supported by the open-ended responses. Nursing students strongly recognized that this IPSE program allowed them to learn essential clinical knowledge in a high-quality simulated and risk-free environment with the opportunity to practice and interact cooperatively. They also commented on the importance of “realistic” training in learning together and the benefits to be realized by breaking down stereotypes and by appreciating both their roles and those of others. The above attitudinal and knowledge improvements indicated in the present study support previous study findings [9, 28]. Stewart and colleagues [9], have demonstrated its effectiveness in improving team-based attitudes and knowledge by conducting IPSE training sessions with high-fidelity simulation among senior medical and nursing students. Sigalet and colleagues [28] expanded training to students from three professions (medicine, nursing, respiratory therapy) in emergency room and intensive care simulation settings and also reported improvements in team-based attitudes.

While effective IPE programs have been shown to have a number of positive benefits, there is still dispute over when to introduce IPE into the academic training of health professionals. Conventional wisdom held that IPE was best offered only after licensure when participants’ professional identities were secure and when they had experiences to share [29, 30]. However, historical evidence [31, 32] shows that IPE should be introduced from the very start of professional education to prevent the formation of negative interprofessional attitudes, which will later be resistant to change. Previous studies have identified fourth-year medical and third-year nursing students as the most appropriate student groups to participate in interprofessional health care education, as they felt confident in their own roles and had the ability to reflect on the roles and contributions of other health care professionals to patient care and clinical practice [19, 33]. In the present study, we recruited third-year nursing students and fourth-year medical students as participants. In accordance with previous studies [9, 28], the results showed that nursing students participating in the study reacted positively to their first IPSE experiences, and had a meaningful positive change in attitudes toward teamwork and collaboration, professional identity, and knowledge about OR nursing. Therefore, the IPSE designated for pre-health professional students may be applied broadly to enhance the effectiveness of team-based training by preparing participants for an interprofessional approach to patient care.

The study limitations must be kept in mind when considering the findings. First, the small sample size limits the ability to generalize findings. The total number of participants, however, does fall within the range of sample sizes for other IPSE experiences in the literature. In addition, the observations were undertaken immediately after the intervention, and it is perhaps not surprising that groups were able to perform to a satisfactory level. To confirm the notion that the continuation of the IPSE program may result in profound changes in post-qualification attitudes, future longitudinal studies are needed. There are logistic constraints in the delivery of this IPSE program, as synchronizing sessions for small groups of students in different professions is not easily achieved. As the number and variety of modules increase and additional professional groups are incorporated, this challenge will increase exponentially.

## Conclusion

We demonstrated that interprofessional training conducted in the simulated OR environment appears to impact positively on participants’ attitudes toward team-based competencies and knowledge of OR nursing. Such an improvement is a key step toward adoption of these attributes within clinical knowledge and skills related to OR nursing for pre-qualifying nursing students. Longer-term follow up using competence for young nursing graduates and healthcare outcomes for patient data may be required to evaluate if short-term benefits among nursing students can translate into improvements in the workplace in the future.

## Abbreviations

OR: Operating room
IPE: Interprofessional education
ICU: Intensive care unit
IPSE: Interprofessional simulation based education
RIPLS: Readiness for Interprofessional Learning Scale
RW: Rongmei Wang
